# Application of Infrared Spectroscopy in Research on Aging of Silicone Rubber in Harsh Environment

**DOI:** 10.3390/polym14214728

**Published:** 2022-11-04

**Authors:** Zhijin Zhang, Tian Liang, Zhenglong Jiang, Xingliang Jiang, Jianlin Hu, Guohui Pang

**Affiliations:** 1 Xuefeng Mountain Energy Equipment Safety National Observation and Research Station of Chongqing University, Chongqing University, Chongqing 400044, China; 2 State Key Laboratory of Disaster Prevention & Reduction for Power Grid Transmission and Distribution Equipment, Disaster Prevention and Reduction Center of State Grid Hunan Electric Power Co., Ltd., Changsha 410007, China

**Keywords:** silicone rubber, FTIR, polymer insulator, material analysis

## Abstract

Polymer insulators using silicone rubber materials as sheds and sheaths are widely used in power systems to replace traditional porcelain and glass insulators which are heavy, inconvenient to install, and prone to pollution flashover. However, in recent years, polymer insulators that have been operating in harsh outdoor environments for many years have experienced different degrees of aging. The aging degree and aging products of silicone rubber are the focus of research. Fourier transform infrared spectroscopy (FTIR) is a technical method to analyze the internal molecular bonds and functional groups of materials, and it is often used to study the aging degree and aging products of silicone rubber. In this paper, the aging characteristics of silicone rubber samples in a high altitude area, salt fog environment, and acid environment were studied by FTIR. The results showed that the silicone rubber in a harsh environment, such as strong radiation, salt fog, and acid fog was degraded to some extent, and its main chain was cut off, the degree of polymerization was reduced, and the content of hydrophobic functional groups was reduced. Infrared spectroscopy can be used to analyze the aging phenomenon of polymers.

## 1. Introduction

Composite insulators using silicone rubber polymer materials as sheds and sheaths have been used in power systems for decades. Compared with more traditional porcelain and glass insulators, they have the advantages of, for example, light weight and easy installation, as well as others. More importantly, the unique hydrophobicity and hydrophobicity transfer of silicone rubber material make it have excellent performance in anti-pollution flashover, so more are increasingly put into new transmission lines [1,2,3,4].

However, the operating environment of the power transmission system is very harsh and complex. Various geographical environmental factors will cause the performance of composite insulators to decline, such as pollution, ultraviolet radiation, acid fog, etc., which may seriously lead to flashover tripping accidents. For example, high energy solar radiation will cut off the molecular bonds in silicone rubber, making its hydrophobicity reduced [5,6]. In this way, the umbrella skirt surface of the composite insulator will be more likely to accumulate a large amount of wet dirt, which will lead to the formation of a dry band [7]. The uneven distribution of water droplets and dirt bands on the surface of silicone rubber will lead to electric field distortion, making the surface more prone to partial discharge. Under the action of electrical aging, silicone rubber polymer materials will undergo irreversible degradation [8,9,10,11]. In coastal areas, the air often contains a large number of sea salt particles, forming a salt-fog environment, and high conductivity salt-fog may cause flashover accidents of power insulators [12]. The research shows that salt-fog will cause partial discharge, damage the silicone rubber material, and reduce its hydrophobicity, and corona aging is more serious than un-energized [13,14,15]. In some areas with heavy industrial pollution, nitrate may be deposited on the insulator surface. At the same time, partial discharge will also promote the reaction of N_2_ and O_2_ in the air to form nitrate [16], pollutants such as salt, nitric acid will affect the performance of silicone rubber [17]. At present, several techniques were applied to study and evaluate the performance of silicone rubber of polymer insulator, such as CTI (Comparative Tracking Index), contact angle, leakage current, and FTIR, etc. For instance, the flashover voltage, or breakdown strength, can reflect the macroscopic electrical insulation property of material. The static contact angle can tell us something about a material’s hydrophobicity. FTIR and scanning electron microscopy (SEM) are often used to study the microstructure of silicone rubber. These methods are often used alone or together to study the properties of the material. [18,19,20,21,22,23].

In recent years, China’s EHV and UHV power energy transmission network is under construction. China’s geographical environment is extremely complex, from the east coast to the hills in the middle, and then to the mountains in the west, long-distance transmission lines will be installed in the harsh climate areas. Outdoor overhead transmission lines will suffer from salt-fog, acid rain caused by industrial pollution, strong solar radiation, and other external factors. Long-term safe and stable operation is the focus of the power industry. In this paper, the infrared spectrum characteristics of silicone rubber samples under three different harsh environments were tested, the content changes of functional groups were determined according to the characteristic peaks of infrared spectrum, and the influence trend of environmental factors on the properties of silicone rubber was analyzed. The results prove that infrared spectroscopy is an effective method for analyzing polymer components.

## 2. Materials and Methods

### 2.1. Samples

The main component of high temperature vulcanized (HTV) silicone rubber (SIR) is polydimethylsiloxane, and a certain proportion of reinforcing agents (silica and silicate) and flame retardants (aluminum hydroxide) are added. The silicone rubber samples in this paper were obtained from a high-altitude strong radiation outdoor environment, a high-humidity salt-fog environment, and a corrosive acid fog environment.

(1) Outdoor high altitude: The test objects are composite insulator samples that have been exposed to the natural environment and aged for different years in the high-altitude area of Xuefeng Mountain, Hunan, with an altitude of 1500 m. These sample insulators are from four different insulator manufacturers and exposed to strong solar radiation in summer, and snow and ice in winter.

(2) Salt-fog environment: Samples for salt-fog treatment were provided by a Chinese composite insulator manufacturer (Xiangfan State Grid Composite Insulator Co., Ltd., Xiangyang, China), and the composition is the same as the composite insulator produced by this manufacturer for operating the transmission line. The salt-fog environment was simulated in the laboratory, and the accelerated aging test was carried out on the silicone rubber. See the following text for the specific test setup.

(3) Corrosive acid-fog environment: Similarly, the original sample are the same as in (2), and the aging samples in this environment are also obtained by simulating the acid-fog environment in the laboratory and performing aging tests.

### 2.2. Aging Experiment and Test Method

**Figure 1:**
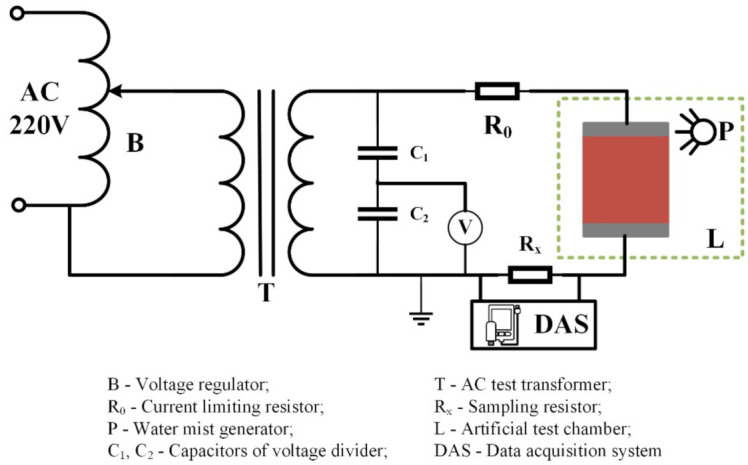
Schematic diagram of device for artificial aging test.

**Figure 2:**
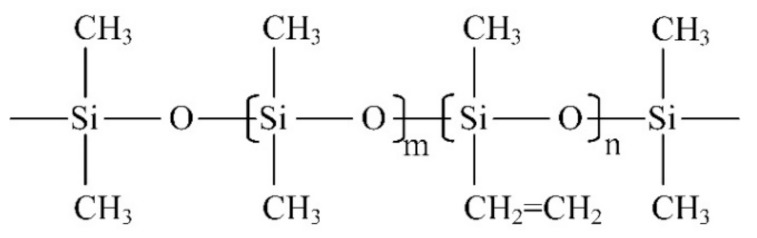
Polydimethylsiloxane structure diagram (m is much greater than n).

(1) Samples preparation: For outdoor long-term natural aging samples, no special treatment is required, and they can be cut into small samples meeting the equipment requirements before testing. For the samples requiring the artificial aging test, in order to ensure that they are not polluted before the test, first clean the surface with alcohol and deionized water, and then put them into the drying oven to dry for 24 h.

(2) Aging process: In this test, the electrical aging of silicone rubber samples in the salt-fog and acid-fog environments were carried out with AC voltage. The experiment platform is shown in Figure 1. During the test, firstly, the salt water or acidic solution were transformed into water mist by an ultrasonic atomizer (Haoqi Electric Appliance Co., Ltd., Zhongshan, China) and transported into the test chamber, and then an AC voltage was applied to the sample to start electrical aging. After 8 h, the experiment was terminated, and the silicone rubber sample was taken out for infrared spectroscopy analysis. Subsequently, the experiment was performed again by changing the conductivity of fog water (γ_20_, means conductivity corrected to 20 °C) and the applied AC voltage (*U_a_*) to compare the influence of environmental factors on the aging degree of silicone rubber.

(3) Fourier transform infrared spectroscopy (FTIR): The Fourier transform infrared spectrometer used in this paper is Nicolet iS50 (Thermo Fisher, Waltham, MA, USA). The spectral measurement range is from 400~4000 cm^−1^, and the resolution is less than 1 cm^−1^. The equipment can be used for transmission and ATR (attenuated total reflection) measurement and is applicable to all kinds of tested products with different phase states. The measurement technique in this paper is ATR, and the resolution is set to 4 cm^−1^. Figure 2 is a schematic diagram of the molecular structure of the main component of the silicone rubber, polydimethylsiloxane (PDMS). The characteristic functional groups in silicone rubber materials and their corresponding infrared wave numbers are shown in Table 1.

## 3. Results and Discussion

In this section, infrared spectrum analysis is carried out for silicone rubber samples in three different environments, the change trend of characteristic functional groups of silicone rubber in different environments is studied, and the ratio of characteristic peaks is used to analyze the change of silicone rubber properties.

### 3.1. Outdoor High Altitude Environment

#### 3.1.1. Infrared Absorption Peak

The silicone rubber samples of the outdoor test station were from four different manufacturers (marked as A–D), and they were all aged for several years in a high-altitude environment, as shown in the Table 2.

**Figure 3:**
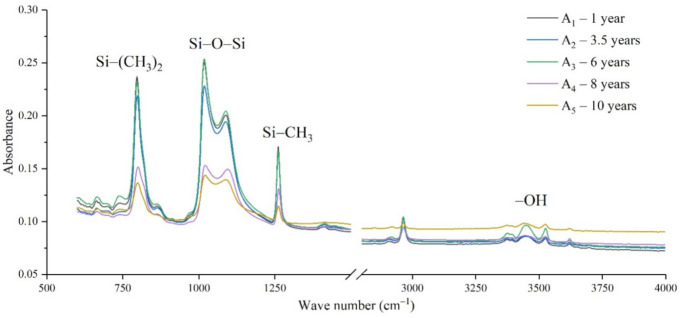
FTIR results of samples from manufacturer A.

**Figure 4:**
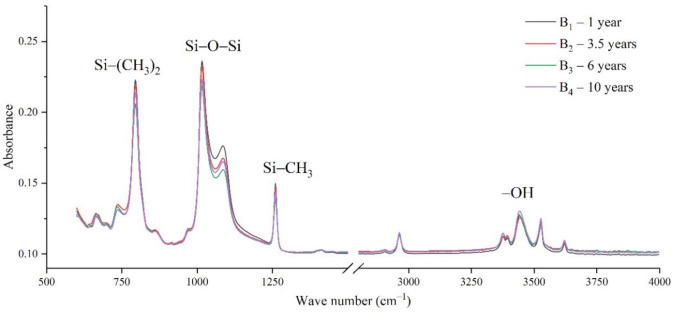
FTIR results of samples from manufacturer B.

**Figure 5:**
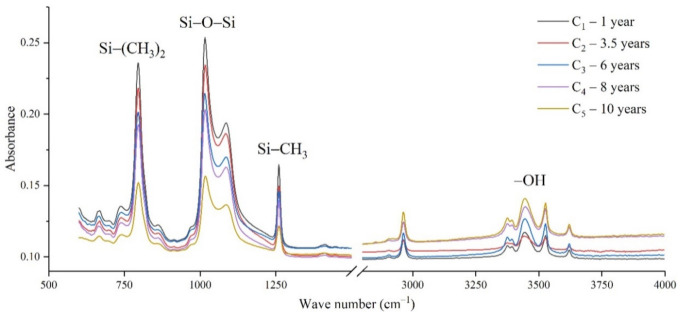
FTIR results of samples from manufacturer C.

**Figure 6:**
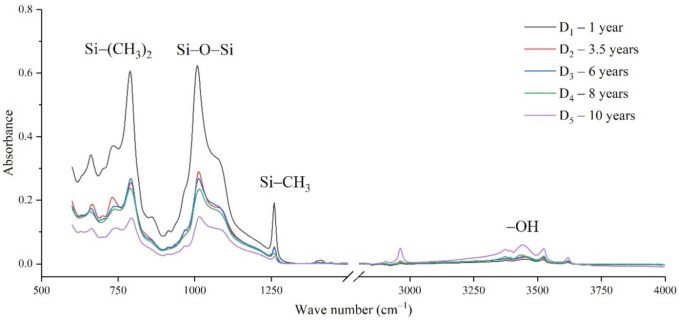
FTIR results of samples from manufacturer D.

The samples in the outdoor test station were all operated without electricity, and the influence of the high-altitude atmospheric environment on silicone rubber was mainly investigated. The chemical structure changes were analyzed by comparing the infrared absorption peak heights of composite insulator silicone rubber samples with a different aging time from four manufacturers. The test results are shown in Figure 3, Figure 4, Figure 5 and Figure 6.

It can be seen from Figure 3, Figure 4, Figure 5 and Figure 6 that the characteristic groups of aged silicone rubber samples at high altitudes have the following characteristics:

(1) The main chain Si-O-Si: Comparing the changes in the absorption peaks of Si-O-Si (1000–1100 cm^−1^) on the surface of the composite insulator silicone rubber samples of four manufacturers with different aging times, it can be concluded that with the increase in aging time, the Si-O-Si absorption peak gradually decreased. This is because in the high-altitude icing environment, the intensity of light and ultraviolet radiation is much stronger than that in the lower-altitude areas, and the composite insulator silicone rubber material is affected by both heat and ultraviolet light. In the long-term aging process, ultraviolet radiation easily breaks the weak parts of the main chain of Si-O-Si, which causes the absorption peak height and area of Si-O-Si to decrease greatly.

(2) C-H bond of side chain: Comparing the changes in C-H bond (1410–1440 cm^−1^) absorption peaks on the surface of composite insulator silicone rubber samples with different aging times from four manufacturers, it can be concluded that with the increase in aging time, the surface C-H absorption peak gradually decreases. The C-H bond is the side bond connected to the main chain of Si-O-Si. The decrease in the C-H absorption peak indicates that the side bond is broken, the number of -CH_3_ groups is reduced, the shielding effect of the long chain of silicone rubber is reduced, and the degree of non-polarity is reduced, resulting in the decrease in material surface hydrophobicity.

(3) -OH group: The hydroxyl group is a hydrophilic group. Comparing the changes in the absorption peak of the -OH group (3200–3700 cm^−1^) on the surface of the composite insulator silicone rubber samples of the four manufacturers with different aging times, it can be concluded that with the increase in aging time, the -OH absorption peak on its surface gradually increased. During the long-term aging process in a high-altitude environment, due to strong light, a variety of free radicals are formed after the main chain is broken, and water molecules combine with it to form hydrophilic silanol groups on the surface of the material.

#### 3.1.2. Ratio *H* of Characteristic Group Absorption Peak Height

Due to the differences in the formula and process of composite insulators from different manufacturers, the height of a single characteristic peak can only represent the change trend of characteristic groups. In this paper, in order to make better use of the measurement results of infrared spectroscopy, in-depth analysis of the differences between the functional groups of samples from different manufacturers, the ratio between the -CH_3_ absorption peak height and the Si-O-Si absorption peak height is used to characterize the chemical aging characteristics of composite insulators from different manufacturers are marked as *H*, namely, *H* is defined as:

$$H=\frac{A_{Si-{\left(CH_{3}\right)}_{2}}}{A_{Si-O-Si}}$$

where $A_{Si-{\left({CH}_{3}\right)}_{2}}$ is the absorption peak height of the side chain and $A_{Si-O-Si}$ is the absorption peak height of the main chain, *H* can be used to characterize the relative content of the side chains, and the higher *H*, the stronger the non-polarity of the silicone rubber.

**Figure 7:**
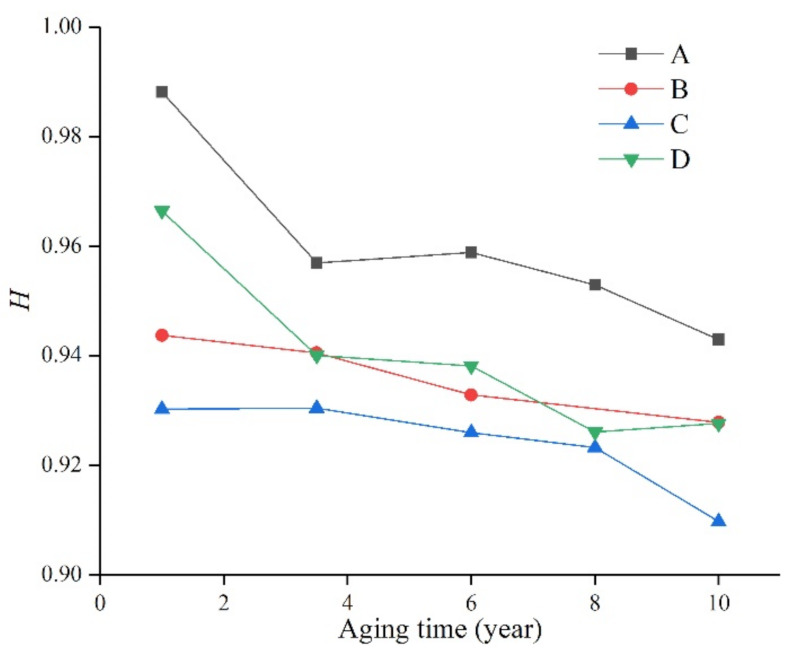
The relationship between *H* and operating time.

The main chain Si-O-Si of the silicone rubber is surrounded and shielded by the hydrophobic group CH_3_, which is also the reason why the silicone rubber has good hydrophobicity. Therefore, the ratio of the absorption peak of Si-(CH_3_)_2_ on the surface of the composite insulator to the absorption peak of the main chain Si-O-Si (that is, *H*) can reflect the relative content of its side bonds, which is related to the degree of non-polarity of the composite insulator. The calculated results of the ratio *H* of the samples are shown in Table 3, Table 4, Table 5 and Table 6 and Figure 7.

It can be seen from Figure 7 that after long-term aging of the silicone rubber samples of the four manufacturers, the *H* value decreased to varying degrees with the increase in aging time, indicating that the surface of the material was damaged and the hydrophobicity decreased.

In addition, due to the different silicone rubber formulations of the manufacturers, the *H* value is still significantly different. For example, the H value of the silicone rubber samples produced by manufacturer A always remained the highest, indicating that its aging resistance is good, and it can still maintain a high level even after ten years of aging. The *H* value of the samples from manufacturer D was similar to that of manufacturer A when they were not aged, but decreased significantly with time. However, the samples from manufacturer C were always at the lowest level, and their performance also decreased with the increase in aging time. From this, it can be seen that the performance of silicone rubber is not only affected by external environmental factors, but also by the production formula and process. Using the ratio of the characteristic peaks in the infrared spectrum, we can study the changes of the properties of silicone rubber more deeply.

### 3.2. Salt-Fog Environment

#### 3.2.1. Infrared Absorption Peak

**Figure 8:**
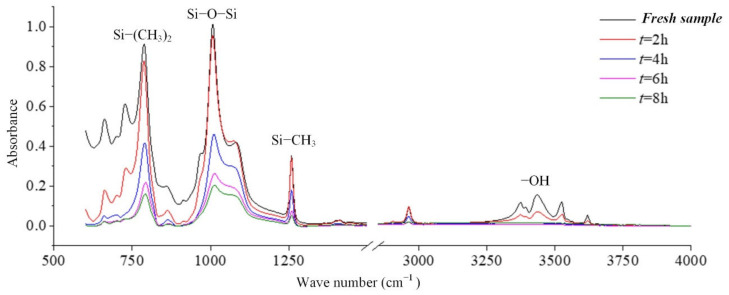
The effect of withstand time on the FTIR spectra (γ_20_ = 1000 μS/cm, AC 3.6 kV).

In coastal areas, the air contains a lot of salt, and these substances will be deposited on the surface of the composite insulator, which increases the leakage current and makes partial discharge more likely to occur. Long-term operation will cause irreversible deterioration of silicone rubber. In this paper, the infrared spectrum analysis of silicone rubber in a salt-spray environment is carried out. Figure 8 is the result of the change in the infrared absorption spectrum of the silicone rubber with the test time [24].

It can be seen from the absorption peaks of the infrared spectrum in Figure 8 that the characteristic peaks Si-(CH_3_)_2_ and Si-CH_3_ of the silicone rubber samples aged in the salt-fog environment are reduced, indicating that the side chain is cut, and the hydrophobic methyl group is reduced. It can be inferred that the static contact angle of the sample surface will become smaller. Similarly, the absorption peaks corresponding to the main chain of Si-O-Si with wave numbers from 1000 cm^−1^ to 1100 cm^−1^ also decreased under the action of salt-fog, indicating that the degree of polymerization decreased. With the extension of test time, the decline trend is more obvious, indicating that the degradation is gradually getting worse [24,25,26].

**Figure 9:**
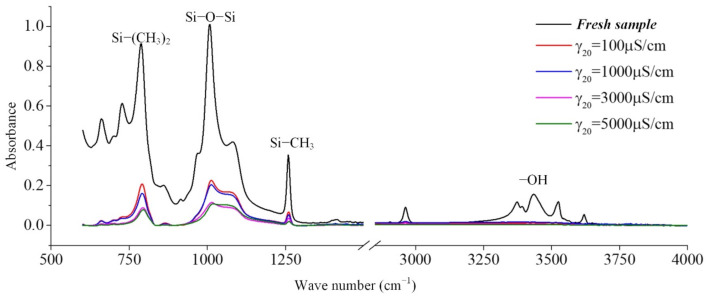
The effect of the water conductivity on the FTIR spectra (*t* = 8 h, AC 3.6 kV).

In the production of silicone rubber, alumina trihydrate (ATH, Al(OH)_3_) is filled into the material for corona resistance and fire retardation, which can reduce temperature and inhibit arc development by consuming ATH. In the infrared spectrum, the region from 3200 cm^−1^ to 3700 cm^−1^ corresponds to the O-H bond in alumina trihydrate and Si-OH. It can be observed from Figure 9 that the absorption peaks in the region from 3200 cm^−1^ to 3700 cm^−1^ are much lower for the samples under the AC voltage under the salt-fog condition compared to the fresh samples, which is different from the uncharged samples in Section 3.1. This shows that in the salt-fog test, ATH in the silicone rubber is decomposed into alumina and water, and the consumption is greater than that of silicone alcohols (Si-OH) generated by molecular bond recombination of silicone rubber. The reason is that in the high-conductivity salt-fog environment, discharge will occur on the surface of the sample, and the high temperature during the discharge process will cause the decomposition of the inorganic flame retardant on the surface of the sample. Thus, the content of OH groups increases due to the recombination of radicals on the one hand and decreases due to the combustion of the arc discharge on the other hand. On the whole, the continuous discharge consumption of ATH dominates, which will also gradually reduce the ablation resistance of silicone rubber. In addition, it is concluded from Figure 9 that under the same aging time, the higher the conductivity of fog water, the more obvious the decrease in the absorption peak.

#### 3.2.2. Ratio *H* of Characteristic Group Absorption Peak Height

As mentioned above, the ratio of the height of the absorption peak of the characteristic functional groups of the side chain and the main chain can be used to study the aging degree of silicone rubber. The *H* value of the sample after artificial aging in the salt-fog environment is shown in Table 7 and Table 8.

It can be seen from Table 7 and Table 8 that the *H* values decrease with the increase in aging time and the increase in fog water conductivity, indicating that the performance of the silicone rubber under charged operation in a long-term salt-fog environment has deteriorated, and its hydrophobicity has decreased. For example, in Table 7, the *H* value of the sample aged for 8 h is 14.1% lower than that of the new sample. In addition, it can be seen from Table 8 that the fog water with very low conductivity (γ_20_ = 100 μS/cm) has almost no effect on the silicone rubber, because during the test there is no discharge on the sample surface, the damage to the sample surface caused by the salt-fog is very small, and the side chain of the silicone rubber is not greatly damaged. Therefore, the *H* value has little difference with the new sample, and even has a slight rise; while, when the conductivity of the fog water increases (γ_20_ = 1000 μS/cm and above), the *H* value drops significantly, indicating that the partial discharge and thermal burning caused by high-conductivity salt-fog are the main reasons for the damage to the surface structure of silicone rubber.

### 3.3. Corrosive Acid-Fog Environment

#### 3.3.1. Infrared Absorption Peak

In areas with serious industrial pollution, composite insulators are likely to operate in a corrosive acid-fog environment. In this paper, we simulate the acid-fog environment in the laboratory, accelerate the aging of silicone rubber samples, and test their infrared spectra to study the performance changes. The test process is as described above, according to the creepage ratio of different pollution degrees [27,28] (very light: 22.0 mm/kV, light: 27.8 mm/kV, medium: 34.7 mm/kV) and the leakage distance of the sample (107 mm), the applied aging voltage *U_a_* was determined to be 4.86 kV, 3.85 kV, and 3.08 kV. The acidic solution is dilute nitric acid solution with pH values of 1.5, 1.9, 2.4, and 2.9, respectively.

**Figure 10:**
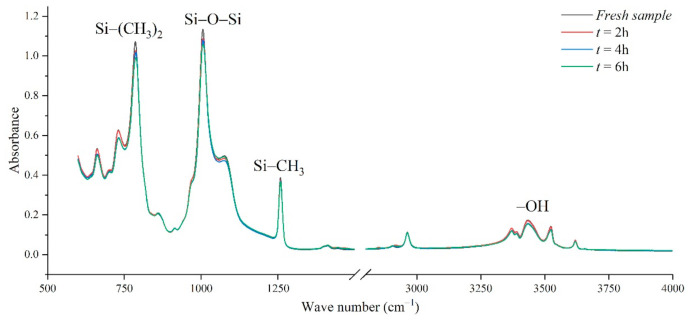
FTIR test results of different test times (AC 3.08 kV, pH = 2.9).

Figure 10 shows the infrared spectrum test diagram of aging samples in a corrosive environment with an aging voltage of 3.08 kV and acid-fog pH of 2.9. Under this test condition, there is no obvious discharge phenomenon on the sample surface. In the figure, the absorption peak strengths of Si-(CH_3_)_2_, Si-O-Si, -OH groups all decrease slowly with the increase in test time.

The nitric acid-fog water covering the sample surface will slowly infiltrate into the silicone rubber matrix. After several hours of acid-fog aging, a new absorption peak representing NO_2_ appears near 1542–1578 cm^−1^, indicating that nitro compounds were produced during the aging process. At the same time, the OH absorption peak in the range from 3700–3200 cm^−1^ also decreased. One way is that nitric acid and ATH neutralized, consuming hydroxyl and generating nitrate. During the nitric acid etching process, the reduction in methyl groups, Si-C bonds, and Si-O-Si structure proves that the PDMS has been cracked. Under the action of chemical equilibrium, on the one hand, strong oxidizing nitric acid combines with the side chain of the broken silicone rubber to generate weak acid, on the other hand, it reacts with the alkaline inorganic filler to generate nitrate. Therefore, the chemical reaction between nitric acid and silicone rubber degrades the polymer and oxidizes to form new groups, causing the material to age.

**Figure 11:**
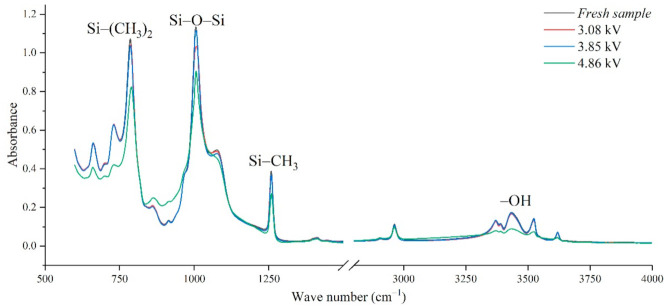
FTIR test results of different applied voltage (pH = 2.4, *t* = 8 h).

**Figure 12:**
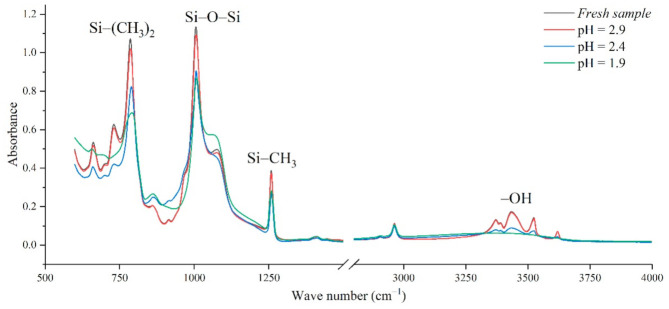
FTIR test results of different pH values (AC 4.86 kV, *t* = 8 h).

In addition, the experimental test results of different aging voltages and different acidity levels are shown in Figure 11 and Figure 12. It can be seen that under the same conditions, the higher the aging voltage value, the lower the pH value of the acidic solution, and the more serious molecular structure of the main chain of silicone rubber is destroyed.

When the test voltage is low (3.08 kV and 3.85 kV), the peak value of the corresponding functional group in the infrared spectrum has little difference, and a slight discharge occurs during the test; while, when the voltage rises to 4.86 kV, the decreasing trend of each functional group becomes obvious. For example, in Figure 11, with the test voltage rising from 3.08 kV to 3.85 kV and 4.86 kV, the Si-O-Si bond decreases by 9.17%, 1.76%, and 20.28%, respectively, compared with the fresh sample. It is concluded that the surface discharge caused by voltage rise will seriously damage the molecular structure of PDMS. In addition, the height of the functional group absorption peak is also related to the acidity of the water mist. With the decrease in pH value, the corrosion damage of acid mist on silicone rubber samples is intensified, and the difference between the infrared spectra of aging samples and fresh samples is increased.

#### 3.3.2. Ratio *H* of Characteristic Group Absorption Peak Height

**Figure 13:**
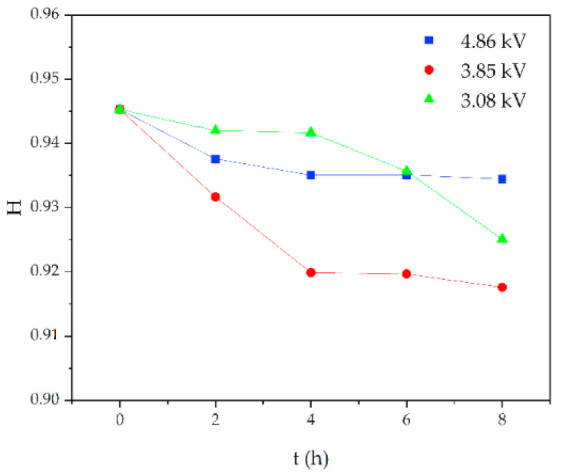
Changes in *H* values of samples with corrosion time (pH = 2.9).

**Figure 14:**
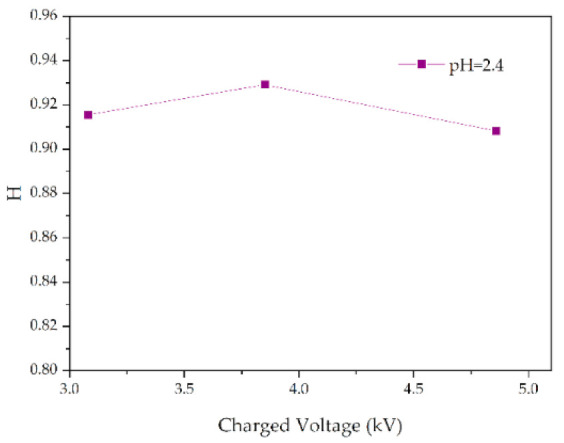
Changes in *H* values of samples with charged voltage (pH = 2.4, *t* = 8 h).

**Figure 15:**
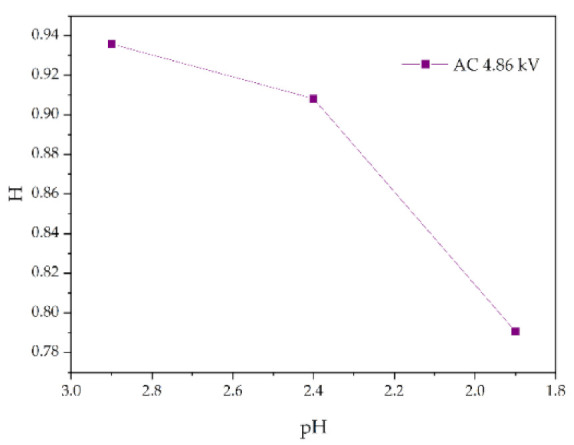
Changes in *H* values of samples with pH value (AC 4.86 kV, *t* = 8 h).

Similarly, for silicone rubber samples aged in the acidic environment, the *H* value is also used to study the relative content of the side chain and main chain to characterize the deterioration degree of silicone rubber. The test results are shown in Figure 13, Figure 14 and Figure 15.

Figure 13 shows the change of *H* value of the tested samples with time under three test voltages. It can be seen that with the increase of corrosion time, the *H* value of samples under different aging voltages decreases gradually. For example, when AC = 3.08 kV, pH = 2.9, the *H* values of 2 h, 4 h, 6 h and 8 h were reduced by 0.35%, 0.38%, 1.02% and 2.14%, respectively, compared with the new samples, indicating that the relative content of the composite insulator side chain is gradually decreasing, the material gradually becomes more polar. It can be seen from Figure 14 that with the increase of voltage, the *H* value decreases, which indicates the fracture of the side chain of the silicone rubber. For example, when pH = 2.4, *t* = 8 h, AC 3.85 kV and 4.86 kV, the *H* value decreased by 1.71% and 3.92%, respectively. Similarly, the *H* value decreases with the decrease of pH value, indicating that the greater the acidity, the more aging of the silicone rubber. For example, when AC 4.86 kV and *t* = 8 h, the h values for pH = 2.9, 2.4 and 1.9 are 0.9358, 0.9082 and 0.7906, respectively, which are 1.00%, 3.92% and 16.37% lower than the fresh samples.

## 4. Conclusions

In this paper, the composite insulator silicone rubber material widely used in the power system is taken as the test object, and the infrared spectrum characteristics of the samples in various environments are studied by using FTIR as the basic analysis method, so as to judge the performance change in the silicone rubber material, which has certain guiding significance for the actual power engineering application.

For the silicone rubber samples aged for many years in the outdoor harsh climate environment, due to the long-term irradiation of ultraviolet rays in high altitude areas and the hydrolysis of water molecules in the air, the main chain of molecules on the surface of silicone rubber is destroyed, the degree of polarity is increased, and a large number of hydrophilic groups are generated on the surface.

The main chain of silicone rubber running with electricity in the salt-fog environment is broken, and the hydrophilic group content of the side chain is reduced. Surface discharge consumes the flame retardant ATH in the material, which weakens the resistance to electric corrosion.

The acid mist environment has corrosive effect on the silicone rubber material running with electricity. The infrared spectrum shows the presence of nitrate, which indicates that the acid mist on the surface of silicone rubber has electrochemical reaction and destroys the microstructure of the material.

Fourier transform infrared spectroscopy can be used to qualitatively analyze the degradation degree of composite silicone rubber in the power system. The height ratio *H* of the characteristic absorption peaks of the side chain and the main chain can characterize the deterioration degree of the silicone rubber surface, and the smaller the value, the more serious the surface damage.

## Figures and Tables

**Table 1:** FTIR absorption bands of HTV SIR material [5,18,20,23].

Functional Group	Wave Number/cm^−1^
Si(CH_3_)_3_	700–800
O-Si(CH_3_)_2_-O	790–840
Si-O-Si	1000–1100
Si-CH_3_	1255–1270
C-H in CH_3_ (bending)	1410–1440
CH in CH_3_ (stretching)	2960–2963
O-H in ATH, Si-OH	3200–3700

**Table 2:** Samples information of composite insulators from different manufacturers.

Aging Years	Samples			
1	A_1_	B_1_	C_1_	D_1_
3.5	A_2_	B_2_	C_2_	D_2_
6	A_3_	B_3_	C_3_	D_3_
8	A_4_	/	C_4_	D_4_
10	A_5_	B_4_	C_5_	D_5_

**Table 3:** The absorption peak height ratio of Si-O-Si and Si(CH_3_)_2_ of composite insulator in A.

Samples	Si-(CH_3_)_2_	Si-O-Si	*H*
A_1_	0.2448	0.2518	0.9881
A_2_	0.2309	0.2413	0.9569
A_3_	0.2189	0.2283	0.9588
A_4_	0.1517	0.1592	0.9529
A_5_	0.1354	0.1436	0.9429

**Table 4:** The absorption peak height ratio of Si-O-Si and Si(CH_3_)_2_ of composite insulator in B.

Samples	Si-(CH_3_)_2_	Si-O-Si	*H*
B_1_	0.2228	0.2361	0.9437
B_2_	0.2198	0.2337	0.9405
B_3_	0.2139	0.2293	0.9328
B_4_	0.203	0.2188	0.9278

**Table 5:** The absorption peak height ratio of Si-O-Si and Si(CH_3_)_2_ of composite insulator in C.

Samples	Si-(CH_3_)_2_	Si-O-Si	*H*
C_1_	0.2363	0.2535	0.9302
C_2_	0.2180	0.2343	0.9304
C_3_	0.2049	0.2213	0.9259
C_4_	0.1876	0.2032	0.9232
C_5_	0.1451	0.1595	0.9097

**Table 6:** The absorption peak height ratio of Si-O-Si and Si(CH_3_)_2_ of composite insulator in D.

Samples	Si-(CH_3_)_2_	Si-O-Si	*H*
D_1_	0.6027	0.6236	0.9664
D_2_	0.2679	0.285	0.9400
D_3_	0.2517	0.2683	0.9381
D_4_	0.2177	0.2351	0.9260
D_5_	0.1384	0.1492	0.9276

**Table 7:** The absorption peak height ratio of Si-O-Si and Si(CH_3_)_2_ (AC 3.6 kV, γ_20_ = 1000 μS/cm).

T (h)	Si-(CH_3_)_2_	Si-O-Si	*H*
Fresh sample	0.914	1.000	0.914
2	0.820	0.956	0.858
4	0.416	0.461	0.902
6	0.219	0.264	0.830
8	0.161	0.205	0.785

**Table 8:** The absorption peak height ratio of Si-O-Si and Si(CH_3_)_2_ (AC 3.6 kV, *t* = 8 h).

γ_20_ (μS/cm)	Si-(CH_3_)_2_	Si-O-Si	*H*
Fresh sample	0.914	1.000	0.914
100	0.208	0.227	0.916
1000	0.161	0.205	0.785
3000	0.090	0.115	0.783
5000	0.079	0.108	0.731

## Data Availability

The data presented in this study are available on request from the corresponding author, upon reasonable request.
